# Mangiferin Exerts Antifungal Activity against *Candida albicans* through Dual Targeting of Cell Wall and Vacuole

**DOI:** 10.4014/jmb.2508.08026

**Published:** 2025-10-28

**Authors:** Younhee Kim

**Affiliations:** Department pf Korean Medicine, Semyung University, Jecheon 27136, Republic of Korea

**Keywords:** Antifungal, β-D-glucan synthase, *Candida albicans*, cell wall, mangiferin, vacuole

## Abstract

Fungal infections caused by *Candida albicans* remain a significant clinical challenge, particularly with increasing resistance to conventional antifungal drugs. The present study aimed to elucidate the antifungal mechanism of mangiferin, a natural polyphenol, against *C. albicans*. Mangiferin was found to impair both cell wall integrity and vacuolar function. Its minimum inhibitory concentration (MIC) was determined to be 156 μg/ml, increasing to 312 μg/ml in the presence of the osmotic protectant sorbitol, suggesting cell wall–specific activity. Fluorescence microscopy with Calcofluor White (CFW), a dye that selectively binds to chitin and β-glucans, revealed weakened staining intensity upon mangiferin treatment, indicating structural alterations of the cell wall. Quantitative analysis showed a 63.9% reduction in CFW fluorescence, consistent with changes in cell wall polysaccharides. Enzymatic assays further showed reduced glucan synthase activity, with (1,3)-β- and (1,6)-β-D-glucans reduced to 82.8% and 94.2%, respectively, confirming inhibition of glucan biosynthesis. In parallel, vacuolar function was compromised. BCECF-AM staining demonstrated diminished vacuolar localization, and ratiometric fluorescence measurements indicated a decrease in intracellular pH from approximately 7.0 to 6.6, reflecting cytosolic acidification and impaired pH homeostasis. Neutral red retention decreased by 20.7%, and morphological observations showed cell enlargement and dye mislocalization, supporting the loss of vacuolar function and osmotic imbalance. Collectively, these findings indicate that mangiferin simultaneously impairs the fungal cell wall and vacuolar systems, leading to compromised turgor maintenance and pH homeostasis. This dual-targeted mechanism underscores mangiferin as a promising multitarget antifungal agent and supports its further development against *Candida* infections.

## Introduction

*Candida albicans* is a major opportunistic fungal pathogen that poses a significant threat to human health, particularly in immunocompromised individuals [1]. As a eukaryotic microorganism, *C. albicans* shares many cellular features with its human host, limiting selective targets for antifungal therapy [2]. Currently available antifungal agents primarily target the fungal cell membrane or cell wall. Azoles disrupt ergosterol, altering membrane structure and function [3, 4]. Polyenes, including amphotericin B, bind directly to ergosterol, causing membrane permeabilization, ion leakage, and cell death [5, 6]. Echinocandins inhibit the synthesis of (1,3)-β-glucan, a key component of the fungal cell wall [7, 8]. Despite their efficacy, these agents have limitations, including resistance and a narrow spectrum of activity, highlighting the need for novel antifungal compounds with distinct mechanisms.

The fungal cell wall is essential for maintaining morphology, structural integrity, and mediating interactions with the host immune system [9]. The yeast cell wall is primarily composed of glycoproteins and polysaccharides, including a mannoprotein layer, a β-1,3-glucan network cross-linked with β-1,6-glucan, and a small amount of chitin. β-glucans constitute approximately 50–60% of the cell wall mass and form the main fibrillar scaffold, while chitin accounts for 1–3% and contributes additional rigidity and resistance to environmental stress. Mannoproteins, comprising 40–50% of the cell wall, shield the glucan layer, maintain hydrophilicity, and influence cell wall porosity and adhesion [10]. β-glucans are synthesized by membrane-associated glucan synthase complexes, whereas chitin is formed by chitin synthases [11]. Inhibition of glucan or chitin biosynthesis disrupts cell wall integrity and can lead to cell death, highlighting these pathways as attractive antifungal targets.

In addition to the cell wall, the fungal vacuole is essential for nutrient storage, detoxification, macromolecule degradation, and ion homeostasis [12]. Proper vacuolar function is vital for survival, as it regulates intracellular pH, morphogenesis, stress responses, and cell wall remodeling. The interplay between vacuolar homeostasis and cell wall biogenesis has emerged as a promising antifungal target [13].

Given the urgent need for novel antifungal agents targeting these critical cellular systems, natural compounds with potential antifungal activity have attracted considerable interest. Among them, mangiferin, a C-glucosyl xanthone primarily isolated from *Mangifera indica*, has drawn attention due to its broad spectrum of pharmacological activities, including antioxidative [14], antimicrobial, antiallergic, antiviral, anticancer, antidiabetic, and antiaging effects [15, 16]. Recent studies have shown that mangiferin can potentiate the activity of conventional antifungal drugs, such as caspofungin and fluconazole, against drug-resistant *Candida* species, and it can additionally enhance efficacy against biofilm-forming strains [17, 18].

*C. albicans* adapts to diverse host environments through dynamic cell wall remodeling, intracellular pH regulation, and vacuolar homeostasis, all of which are critical for survival and pathogenicity. Given the growing resistance to conventional antifungal drugs, compounds targeting multiple cellular systems have attracted increasing interest. The present study evaluates the antifungal activity of mangiferin against *C. albicans*, focusing on its effects on cell wall integrity and vacuolar function, and highlights its potential as a multitarget antifungal agent.

## Materials and Methods

### Microorganisms

*C. albicans* SC5314, *C. krusei* ATCC 6258 (KCCM 11426), *C. glabrata* ATCC 2001 (KCCM 50044), and *C. parapsilosis* ATCC 22019 were used in this study. Strains were obtained from the American Type Culture Collection (ATCC, USA) and the Korean Culture Center of Microorganisms (KCCM, Republic of Korea). *C. albicans* SC5314 was primarily used for all experimental procedures, while the other strains were included for comparative purposes.

### Reagents

Mangiferin, dimethyl sulfoxide (DMSO), propidium iodide (PI), and Calcofluor White (CFW), neutral red, protease inhibitor cocktail, sorbitol, and amphotericin B were purchased from Sigma-Aldrich (USA). CFDA-AM, BCECF-AM, RPMI-1640 medium, and phosphate-buffered saline (PBS, pH 7.2) were obtained from Invitrogen (USA). Mangiferin stock solutions were prepared in DMSO and stored at -20°C in the dark. All other reagents were of analytical grade.

### Antifungal Susceptibility Testing

The antifungal activity of mangiferin against *Candida* strains was assessed using a broth microdilution assay following CLSI M27-A3 guidelines [19], with 0.1 mg/ml resazurin as a viability indicator [20]. Two-fold serial dilutions of mangiferin or amphotericin B (100 μl) were prepared in RPMI-1640 medium in a 96-well plate, and 100 μl of yeast suspension (1 × 10^3^–5 × 10^3^ cells/ml) was added. Plates were incubated at 35°C for 48 h, and MICs were determined colorimetrically as the lowest concentration at which the color remained blue. DMSO served as a solvent control, and amphotericin B as a positive control.

### Sorbitol Protection Assay

The sorbitol protection assay was performed following CLSI M27-A3 guidelines with minor modifications [21]. *C. albicans* inocula were prepared to a final cell density of 1 × 10^3^–5×10^3^ cells/ml to ensure reproducibility. Two-fold serial dilutions of mangiferin were prepared in RPMI-1640 medium with 0.1 mg/ml resazurin, in the absence or presence of 0.8 M sorbitol in separate rows of a 96-well round-bottom microplate. *C. albicans* cell suspensions were added to each well, and MIC values were determined after 48 or 72 h of incubation at 35°C.

### Triple Staining for Viability, Membrane Integrity, and Cell Wall Structure

A triple-staining protocol using CFDA-AM, PI, and CFW was employed to assess the effects of mangiferin on *C. albicans* cell viability, membrane integrity, and cell wall structure. Log-phase *C. albicans* cells (1 × 10^7^ cells/ml) were treated with mangiferin at a sub-inhibitory concentration (0.5× MIC) or 0.26% DMSO as a control at 35°C for 2.5 h. Cells were then stained with 10 μg/ml CFDA-AM, 10 μg/ml PI, and 0.025% CFW for 30 min at 30°C in the dark prior to fluorescence microscopy.

### CFW Binding Assay

To assess cell wall polysaccharide content, including chitin and b-glucans, CFW binding was measured. Exponential-phase *C. albicans* cells (2 × 10^7^ cells/ml) were pre-stained with 0.002% CFW to establish a uniform baseline. Mangiferin (0.5×, 1×, or 2× MIC) or DMSO control was added, and cells were transferred to a black 96-well microplate. Following a 7 min setup period for plate preparation, fluorescence readings were recorded at 7, 10, 20, 30, and 40 min using a microplate reader (Infinite M200 Pro, Tecan, Austria) with excitation at 360 ± 35 nm and emission at 485 ± 20 nm. Relative CFW binding at 20 min was expressed as a percentage of the DMSO control. Data represent one of three independent replicates.

### Microsomal Membrane Preparation

Microsomal fractions were prepared from *C. albicans* SC5314 using a previously reported method [22], adapted from the protocol by Shedletzky *et al*. [23], with the exception of cell disruption: Harvested cells were suspended in ice-cold breakage buffer containing 50 mM Tris-Cl (pH 7.4), 1 mM EGTA, 20 μM PMSF, 4 mM DTT, and 5 μM leupeptin, and then disrupted using Lysing Matrix C (1.4 mm zirconia and silica beads) and a FastPrep-24 homogenizer (MP Biomedicals, USA). All subsequent steps followed the protocol described in [22].

### Protein Quantification

Protein concentration of the microsomal fraction was determined using the Bradford assay (Bio-Rad, USA) with bovine serum albumin as a standard, yielding approximately 4 mg/ml.

### Glucan Synthase Activity Assay

The enzymatic activities of (1,3)-β-D-glucan synthase and (1,6)-β-D-glucan synthase were measured using microsomal membranes and quantified by the aniline blue assay, based on modified procedures from Shedletzky *et al*. [22, 23] and Vink *et al*. [24], respectively. Each 50 μl reaction contained 32 μg of microsomal protein and was initiated by adding mangiferin at the indicated concentrations, followed by incubation at 25°C for 50 min. The (1,3)-β-D-glucan mixture contained 50 mM Tris-HCl (pH 7.5), 5 mM EDTA, 0.8% Brij 35, 20% glycerol, 2 mM UDP-glucose, and 20 μM GTP. The (1,6)-β-D-glucan mixture contained 50 mM sodium citrate (pH 6.5), 1% BSA, 1 mM EDTA, 20% glycerol, 2.5 mM UDP-glucose, and 150 μM GTP. Reactions were terminated with 10 μl of 6 N NaOH and heating at 80°C for 30 min. Glucan production was quantified by adding freshly prepared aniline blue reagent and incubating at 50°C for 30 min, followed by cooling to room temperature for 30 min. Aliquots were transferred in quintuplicate to a black 96-well microplate, and fluorescence was measured using a microplate reader. Excitation/emission wavelengths were 400/485 nm for (1,3)-β-glucan and 400/520 nm for (1,6)-β-glucan. Representative data are shown.

### BCECF-AM Staining and Fluorescence Microscopy

The pH-sensitive dye BCECF-AM was used to assess intracellular pH in logarithmic-phase *C. albicans*. Cells were treated with mangiferin (1× MIC) or DMSO (solvent control) at 35°C for 4 h, then labeled with 10 μM BCECF-AM in PBS (pH 7.2) and incubated at 30°C for 30 min in the dark. Heat-killed cells (85°C, 10 min) served as a control. Bright-field and fluorescence images were acquired using a fluorescence microscope with a GFP filter set (Olympus IX-51) and captured with CellSens software (Olympus, Japan).

### Intracellular pH Calibration and Measurement of Mangiferin-Induced Changes in pH_i_

Intracellular pH (pH_i_) was determined using the ratiometric fluorescent dye BCECF-AM, with modifications from a previous study [25]. Logarithmic-phase *C. albicans* cells (1 × 10^7^ cells/ml) were washed twice with distilled water and incubated in calibration buffers (pH 4.5, 5.5, 6.5, and 7.2) containing 10 μM BCECF-AM, 10 μM valinomycin, and 10 μM nigericin at 35°C for 1 h in the dark with gentle agitation. Fluorescence emissions at 485 nm and 460 nm (bandwidth 20 nm) were measured separately in a black 96-well microplate, and the ratio (I_485_/I_460_) was used to generate a standard curve.

To evaluate the effect of mangiferin on pH_i_, *C. albicans* cells (1 × 10^7^ cells/ml) were treated with mangiferin at 0, 1×, 2×, and 3× MIC in PBS (pH 7.2) and incubated at 35°C for 1 h. Cells were then washed twice with PBS (pH 7.2), loaded with 10 μM BCECF-AM in PBS (pH 7.2) for 30 min in the dark, and measured under the same fluorescence settings as the calibration. The resulting fluorescence ratios were converted to intracellular pH values using the calibration curve. The regression line was obtained from four independent experiments, each measured in five replicate wells (*n* = 5). To enhance visualization of the ratio values, the y-axis range of the standard curve and intracellular pH plots was adjusted to 0.7–1.1.

### Neutral Red Retention Assay for Vacuolar Function

Vacuolar function of *C. albicans* was assessed using a neutral red retention assay, modified from the neutral red uptake assay [26]. Cells in exponential phase were washed with PBS (pH 7.2) and incubated with 0.03% neutral red at 35°C for 30 min with mild shaking. After washing, cells were adjusted to 1 × 10^6^ cells/ml and treated with mangiferin (0.5×, 1×, or 1.5× MIC) or an equivalent volume of DMSO as control at 30°C for 30 min in the dark. Cells were harvested, washed, and resuspended in extraction buffer (1% acetic acid, 50% ethanol, 49% water) for 20 min at room temperature. Aliquots were transferred in quadruplicate to a black 96-well microplate, and fluorescence was measured (excitation 535 nm, emission 635 nm, ±20 nm).

### Staining with Neutral Red

Following the neutral red retention assay, vacuolar function was further evaluated by fluorescence imaging. *C. albicans* cells were treated with 1× MIC mangiferin or an equivalent volume of DMSO for 2 h. After treatment, cells were washed and incubated with 0.025% neutral red for 20 min in the dark. Cells were washed again and immediately observed by bright-field and fluorescence microscopy.

### Statistical Analysis

All experiments were independently repeated at least twice, with each condition measured in quadruplicate or quintuplicate. Data are presented as mean ± SD. Statistical significance was assessed using unpaired two-tailed *t*-tests or one-way ANOVA followed by Tukey’s post hoc test, as appropriate. Analyses were performed using SigmaPlot 13.0 (Systat Software Inc., USA), and *p* values < 0.05 were considered statistically significant.

## Results

### Mangiferin Exhibits Antifungal Activity against *Candida* spp.

The antifungal potential of mangiferin was evaluated by determining the minimum inhibitory concentrations (MICs) against four *Candida* species of *C. albicans*, *C. krusei*, *C. glabrata*, and *C. parapsilosis* (Table 1). Mangiferin exhibited MIC values of 156 μg/ml, 32 μg/ml, 8 μg/ml, and 64 μg/ml against these strains, respectively. In comparison, amphotericin B showed MIC values of 1 μg/ml, 0.5 μg/ml, 1 μg/ml, and 1 μg/ml, respectively. These results indicate that mangiferin possesses broad-spectrum antifungal activity, with relatively higher efficacy against *C. glabrata*. Based on its clinical significance and extensive characterization as a fungal model organism, *C. albicans* was selected for subsequent mechanistic studies.

### Mangiferin Disrupts Cell Wall Integrity in *C. albicans* as Assessed by Sorbitol Protection Assay

The effect of mangiferin on the fungal cell wall was assessed using a sorbitol protection assay with *C. albicans* incubated in the presence or absence of 0.8 M sorbitol as an osmotic stabilizer. After 48 h of incubation, the MIC of mangiferin remained unchanged at 156 μg/ml regardless of sorbitol presence (Table 2). However, after 72 h, the MIC increased from 156 μg/ml (without sorbitol) to 312 μg/ml (with sorbitol). This delayed increase in MIC suggests that mangiferin may gradually impair cell wall integrity.

### Mangiferin Reduces Cell Viability and Alters Cell Wall Structure in *C. albicans* as Revealed by Triple Staining

**Fig. 1:**
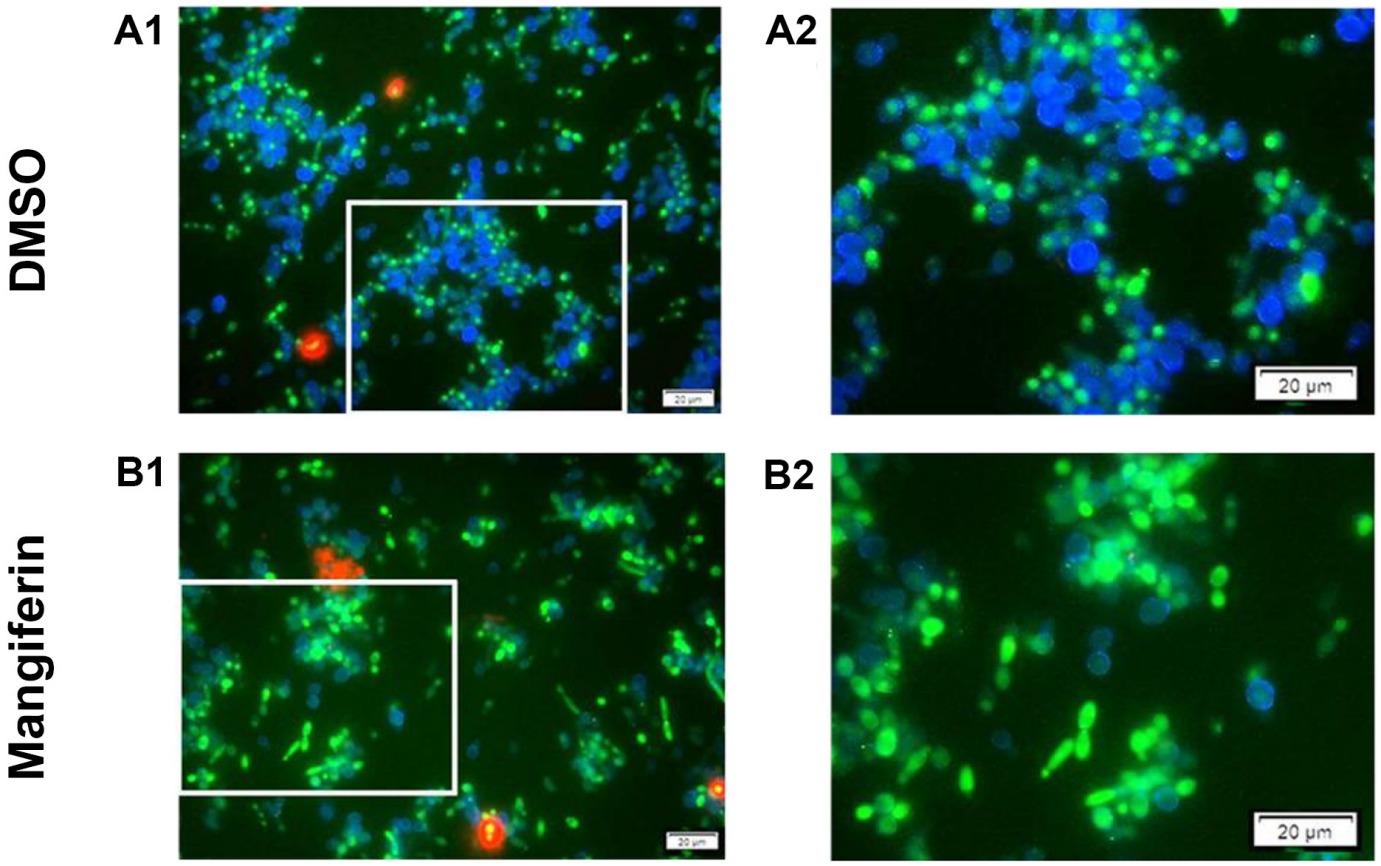
Fluorescence staining of *C. albicans*. Cells were treated with 78 μg/ml mangiferin (0.5× MIC) or 0.26% DMSO for 2.5 h, followed by triple staining with CFDA-AM (green), PI (red), and CFW (blue). Images were captured using an RGB filter set under identical exposure conditions. Panels A2 and B2 are enlarged views of the boxed areas in A1 and B1. Scale bar: 20 μm.

To assess the effects of mangiferin on cell viability, membrane integrity, and cell wall structure, *C. albicans* cells were stained with CFDA-AM, PI, and CFW, and visualized under fluorescence microscopy using identical exposure settings (Fig. 1). CFDA-AM is a cell-permeant, lipophilic esterase substrate that is hydrolyzed by intracellular esterases to yield the green fluorescent compound carboxyfluorescein. The product, being negatively charged, is retained in the cytoplasm of live cells and serves as a viability probe. CFDA staining showed comparable levels of green fluorescence between the DMSO control group (Fig. 1A1 and Fig. 1A2) and the mangiferin-treated group (Fig. 1B1 and Fig. 1B2), suggesting that mangiferin at 0.5× MIC does not significantly impair cell viability. Although the green signal in A2 was partially masked by intense blue fluorescence from CFW, the green fluorescence in B2 appeared more prominent in some cells due to an increase in cell size, with several cells also showing altered staining patterns. Such differences may reflect early morphological alterations induced by mangiferin. The membrane integrity indicator PI, which penetrates yeast cells with compromised membranes and emits red fluorescence upon binding to DNA, revealed a slight increase in red signal in mangiferin-treated cells (Fig. 1B1) compared to the control (Fig. 1A1), indicating minor membrane damage. A slight elevation in PI-positive cells was also observed in the 0.26% DMSO-treated group, consistent with the known membrane-disruptive potential of DMSO. Nevertheless, the overall proportion of PI-stained cells remained low in both groups, suggesting that neither mangiferin nor DMSO caused substantial membrane disruption. In contrast, a clear difference was observed in CFW staining, which binds to chitin [27] and β-glucans [28] in the fungal cell wall. Control cells (A1 and A2) exhibited strong blue fluorescence, indicating the presence of intact and abundant CFW-binding polysaccharides. In mangiferin-treated cells (Fig. 1B1 and Fig. 1B2), however, blue fluorescence was markedly diminished, with only a few cells displaying faint staining. These observations suggest that mangiferin may alter the structure, composition, or accessibility of cell wall polysaccharides, thereby supporting the hypothesis that the cell wall is a potential target of its antifungal activity.

### Mangiferin Affects Cell Wall Architecture in *C. albicans* as Revealed by CFW Staining

**Fig. 2:**
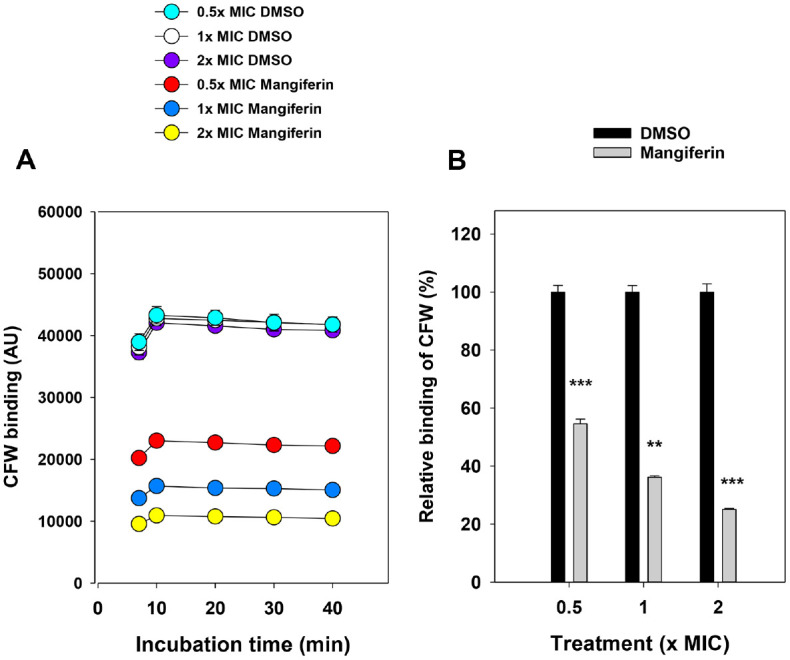
CFW binding in *C. albicans*. (**A**) Time-course of CFW fluorescence intensity (arbitrary units, AU) in cells treated with mangiferin at 0.5×, 1×, and 2× MIC or an equivalent volume of DMSO. Six replicate wells per condition. Excitation/ emission: 360/485 nm. (**B**) Relative CFW binding (%) at 20 min, normalized to DMSO control (100%). Bars represent mean ± SD. Statistical significance determined by unpaired two-tailed *t*-tests (**p < 0.01, ***p < 0.001).

To evaluate the impact of mangiferin on cell wall polysaccharides, including chitin and β-glucans, CFW binding was monitored over time following treatment with subinhibitory to inhibitory concentrations of mangiferin. Fluorescence intensity in all samples peaked around 10 min and gradually decreased thereafter, likely due to photobleaching of CFW fluorescence. Fluorescence was consistently higher in DMSO-treated cells than in any mangiferin-treated group (Fig. 2A). Quantitative analysis of relative fluorescence at the 20-min time point (Fig. 2B) confirmed this trend. Compared to the DMSO control, mangiferin-treated cells exhibited significantly reduced CFW binding: 54.6% at 0.5× MIC, 36.1% at 1× MIC, and 25.0% at 2× MIC (*p* < 0.01). The contrast in CFW staining patterns observed by fluorescence microscopy between DMSO- and mangiferin-treated cells (Fig. 1) aligns with these quantitative results, supporting the conclusion that mangiferin compromises cell wall organization. These findings suggest that mangiferin induces structural alterations in the fungal cell wall, potentially affecting the composition or accessibility of chitin and β-glucans, thereby diminishing CFW binding capacity.

### Mangiferin Inhibits (1,3)- and (1,6)-β-D-Glucan Synthase in *C. albicans*

**Fig. 3:**
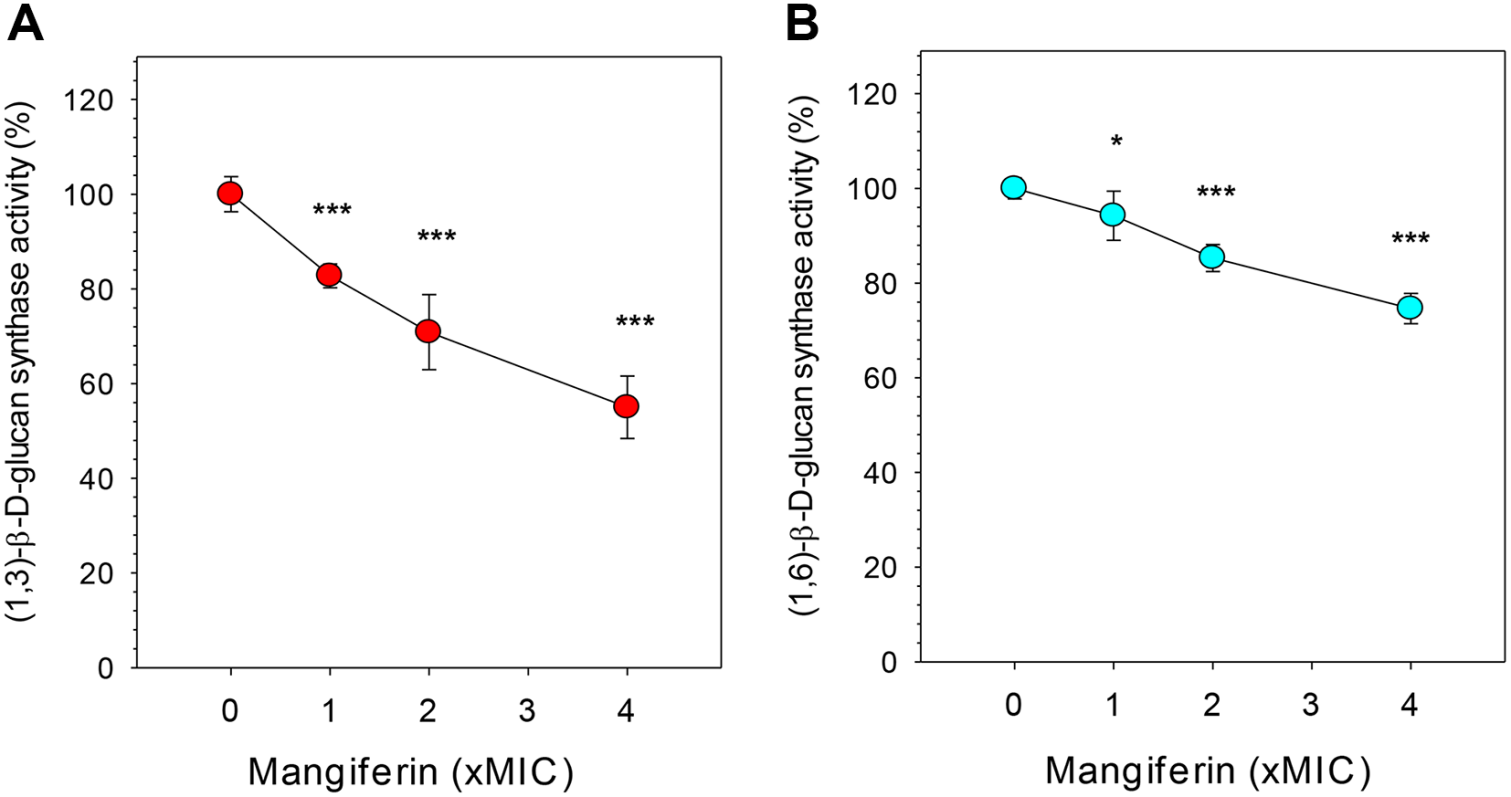
Microsomal glucan synthase activity in *C. albicans*. Microsomal membranes were incubated with the indicated concentrations of mangiferin. Activities of (**A**) (1,3)-β-D-glucan synthase and (**B**) (1,6)-β-D-glucan synthase were determined by aniline blue fluorescence under their respective assay conditions. Fluorescence intensity was recorded at excitation/emission wavelengths of 400/485 nm for (1,3)-β-D-glucan, and 400/520 nm for (1,6)-β-D-glucan. Bars represent mean ± SD of technical replicates. Statistical significance: unpaired two-tailed *t*-tests (*p < 0.05, ***p < 0.001).

To evaluate whether mangiferin disrupts fungal cell wall biosynthesis, the activities of (1,3)-β-D-glucan synthase and (1,6)-β-D-glucan synthase were measured in microsomal membrane fractions of *C. albicans* treated with varying concentrations of mangiferin. As shown in Fig. 3, mangiferin significantly inhibited both (1,3)-β-D-glucan and (1,6)-β-D-glucan synthase activities in a concentration-dependent manner. Compared to the untreated control, (1,3)-β-D-glucan synthase activity decreased to 82.8 %, 70.9%, and 55.0 % at 1×, 2×, and 4× MIC concentrations of mangiferin, respectively. Similarly, (1,6)-β-D-glucan synthase activity was reduced from 100% in the control to 94.2%, 85.3%, and 74.6% at the corresponding mangiferin concentrations. These results indicate that mangiferin effectively inhibits glucan synthase activities, suggesting that interference with cell wall biosynthesis is a key component of its antifungal mechanism. This enzymatic inhibition is in line with the reduced CFW fluorescence observed in both microscopic staining (Fig. 1) and quantitative binding assays (Fig. 2), which reflect compromised cell wall architecture. Together, these findings suggest that mangiferin disrupts fungal cell wall integrity by impairing the synthesis and organization of major polysaccharide components, including chitin and β-glucans.

### Mangiferin Impairs Vacuolar Function and Alters Intracellular pH in *C. albicans* as Revealed by BCECF-AM Fluorescence

**Fig. 4:**
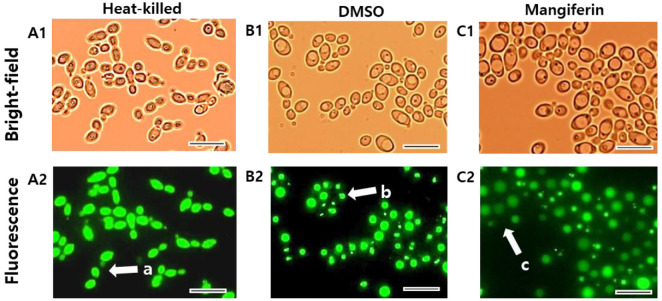
BCECF-AM fluorescence microscopy of *C. albicans*. Top row shows bright-field images; bottom row shows corresponding fluorescence images. Columns from left to right represent heat-killed cells (**A1, A2**), DMSO-treated control cells (**B1, B2**), and mangiferin-treated cells (**C1, C2**). Regions a–c indicate representative areas. Images captured using a GFP filter set. Scale bar: 10 μm.

In addition to its effects on the cell wall, mangiferin also affected vacuolar function, a key aspect of fungal physiology. Effect of mangiferin on intracellular pH and compartmentalization was assessed by BCECF-AM staining and fluorescence microscopy. In heat-killed cells (Fig. 4A1 and 4A2), cell morphology was retained in the bright-field image, while BCECF fluorescence was diffusely distributed throughout the cytoplasm, indicating loss of compartmentalization (region a; Fig. 4A2). DMSO-treated control cells (Fig. 4B1 and 4B2) showed BCECF fluorescence predominantly localized to discrete vacuoles, and a cluster of cells (region b) exhibited strong green fluorescence confined to vacuoles, consistent with proper pH compartmentalization. By contrast, mangiferin-treated cells (Fig. 4C1 and 4C2) showed heterogeneous fluorescence patterns with reduced overall signal intensity, indicative of impaired vacuolar acidification and compromised vacuolar function.

### Mangiferin Disrupts Intracellular pH Homeostasis in *C. albicans*

**Fig. 5:**
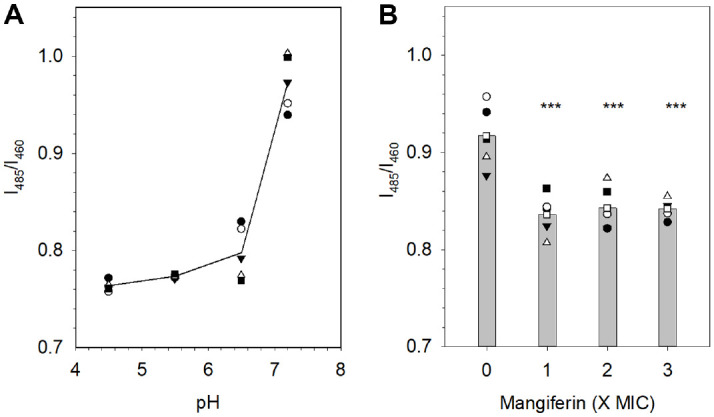
Effects of mangiferin on intracellular pH of *C. albicans*. (**A**) Standard curve for ratiometric calibration of intracellular pH using BCECF-AM, with regression line and five replicate wells (*n* = 5) from four independent experiments. (**B**) Intracellular pH values after mangiferin treatment, shown as bars with six individual data points (*n* = 6) overlaid. Error bars were removed for clarity, and the y-axis range was adjusted to 0.7–1.1 to enhance visibility of the data. Statistical significance compared with control is indicated as *** *p* < 0.001.

Intracellular pH (pH_i_) was quantitatively measured using the ratiometric fluorescent dye BCECF-AM, which exhibits pH-dependent excitation shifts [29]. A calibration curve was generated by plotting fluorescence ratios of BCECF-AM–stained *C. albicans* cells equilibrated in buffers of known pH values (4.5, 5.5, 6.5, and 7.2), as shown in Fig. 5A. The fluorescence ratio increased with pH, ranging from 0.76 at pH 4.5 to 0.97 at pH 7.2. The resulting curve displayed a gradual increase between pH 4.5 and 6.5, followed by a sharper rise at pH 7.2, which reflects the dye’s sensitivity near neutral pH. As shown in Fig. 5B, treatment with mangiferin at 1×, 2×, and 3× MIC concentrations resulted in significantly lower I_485_/I_460_ fluorescence ratios compared to the DMSO-treated control. Based on the calibration curve, control cells exhibited a pH_i_ of approximately 7.0, whereas mangiferin-treated cells showed slightly reduced pH_i_ values of 6.6–6.7. These quantitative results corresponded with the imaging data, reflecting impaired vacuolar acidification and disrupted pH compartmentalization. Collectively, these findings demonstrate that mangiferin compromises vacuolar pH homeostasis in *C. albicans*.

### Mangiferin Compromises Vacuolar Function in *C. albicans* as Assessed by Neutral Red Retention Assay

**Fig. 6:**
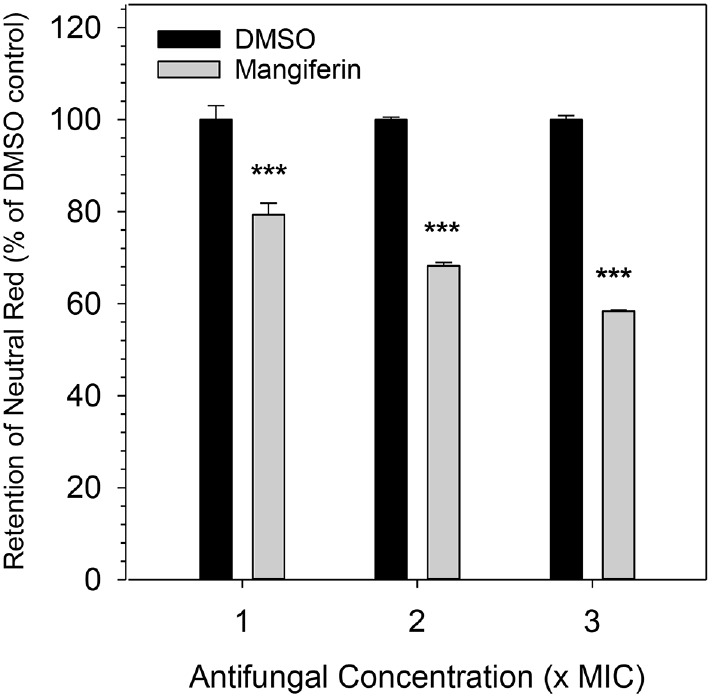
Neutral red retention assay for vacuolar function in *C. albicans*. Cells were preloaded with neutral red, washed to remove excess dye, and treated with mangiferin at 1×, 2×, or 3× MIC, or an equivalent volume of DMSO. After 30 min, intracellular dye was extracted and fluorescence measured at excitation/emission 535/635 nm. Fluorescence values are expressed relative to DMSO control (100%). Bars represent mean ± SD of four technical replicates. Statistical significance: unpaired two-tailed *t*-tests (***p < 0.001).

Neutral red is a weak basic dye that readily diffuses across membranes in its uncharged form. It accumulates selectively in acidic compartments such as vacuoles through protonation and trapping, and its retention serves as an indirect indicator of vacuolar membrane integrity [26]. When vacuolar pH or membrane integrity is disrupted, neutral red may leak back into the cytosol in its unprotonated form, leading to decreased dye retention. To investigate whether the observed intracellular acidification is associated with vacuolar dysfunction, a neutral red retention assay was performed (Fig. 6). Compared with the efficient neutral red retention observed in vacuoles of DMSO-treated control cells, mangiferin-treated cells exhibited a dose-dependent decrease in dye retention, with fluorescence intensity reduced to 79.3%, 68.2%, and 58.3% at 1×, 2×, and 3× MIC, respectively (*p* < 0.001). These results reveal that mangiferin impairs vacuolar function in *C. albicans*.

### Microscopic Observation Confirms Vacuolar Dysfunction in *C. albicans* Using Neutral Red Staining

**Fig. 7:**
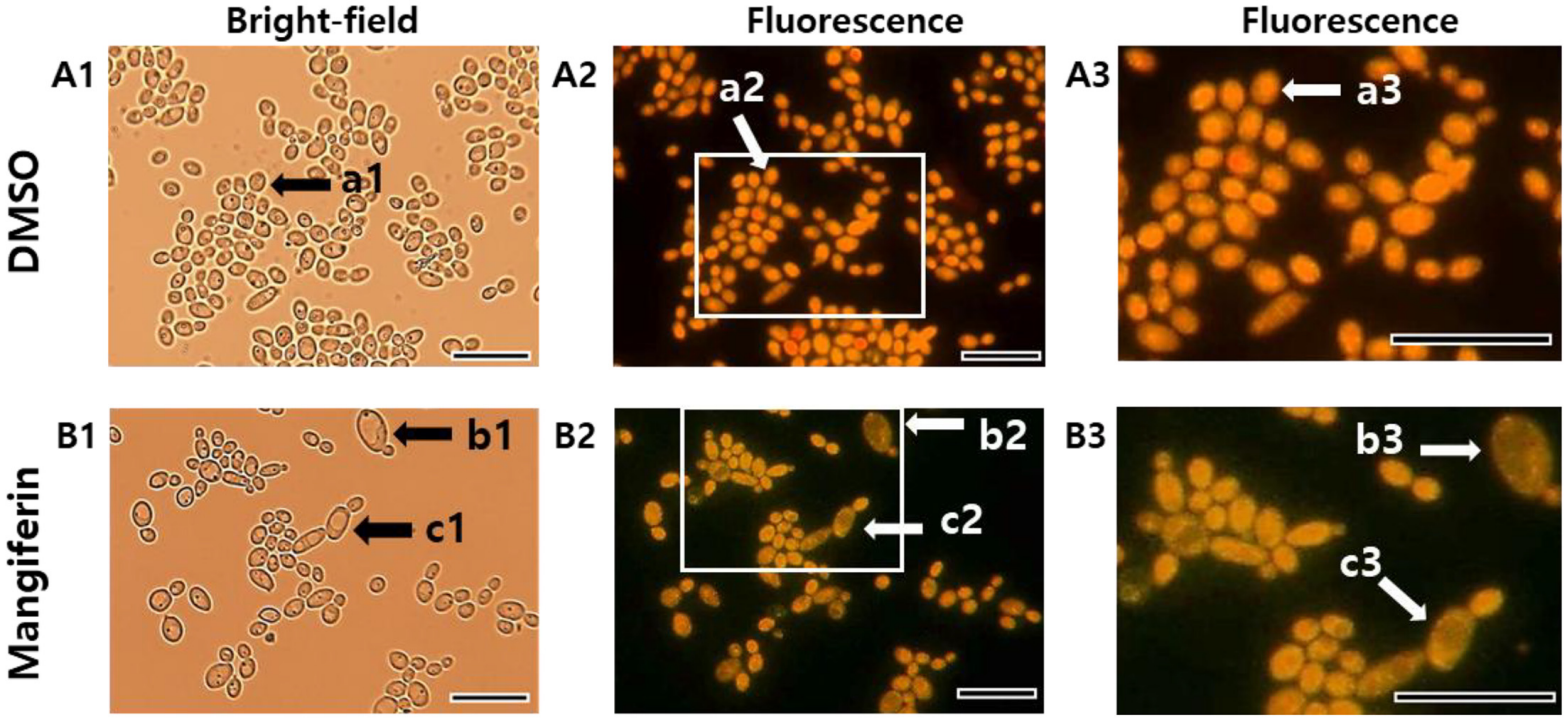
Neutral red staining of *C. albicans*. Cells were treated with DMSO (**A1–A3**) or mangiferin (**B1–B3**) for 2 h, stained with 0.025% neutral red in PBS (pH 7.2) for 20 min, and imaged by bright-field (**A1, B1**) and fluorescence microscopy (**A2, A3, B2, B3**). Panels A3 and B3 show magnified views of boxed regions in A2 and B2. Arrow-labeled cells indicate the same cells across panels. Scale bar: 20 μm.

To examine vacuolar morphology and dye localization at the single-cell level, cells were stained with neutral red and observed under bright-field and fluorescence microscopy. In DMSO-treated cells (Fig. 7A1–7A3), the dye accumulated prominently in vacuoles (a3), reflecting intact acidification. In contrast, mangiferin-treated cells (Fig. 7B1–7B3) exhibited altered morphology, often appearing swollen and enlarged compared to typical controls. Although vacuoles remained visible under bright-field microscopy (b1, c1), neutral red retention was markedly reduced, with weak red signals frequently redistributed to the cytoplasm and displaced from the vacuolar region (b3, c3). These findings corroborate dose-dependent vacuolar dysfunction induced by mangiferin. Collectively, these findings suggest that mangiferin induces intracellular acidification and compromises vacuolar integrity, which may contribute to its antifungal activity by disrupting organelle function and pH homeostasis.

## Discussion

The sorbitol protection assay indicated that osmotic stabilization partially mitigated mangiferin’s inhibitory effect on *C. albicans*, suggesting direct interference with cell wall integrity. CFW binding, which monitors chitin and β-glucans [27, 28], revealed that mangiferin significantly reduced the fluorescence signal from cell wall polysaccharides compared with DMSO-treated controls. Rapid reduction in fluorescence intensity within minutes of treatment supports the conclusion that mangiferin interferes with structural polysaccharides rather than merely reflecting cell death. Since β-glucans constitute the major fibrillar framework of the *Candida* cell wall [10,30], the observed reduction in glucan synthase activity in microsomal assays provides strong evidence that mangiferin impairs polysaccharide biosynthesis and weakens cell wall integrity.

Concurrently, vacuolar function was disrupted by mangiferin, as evidenced by BCECF-AM fluorescence imaging and ratiometric pH measurements. BCECF-AM, a pH-sensitive fluorescent dye that localizes predominantly in the vacuole of *C. albicans* [31], revealed that treated cells exhibited heterogeneous vacuolar morphology and reduced pH_i_ values, indicating impaired vacuolar acidification. The neutral red retention assay provided complementary evidence. Unlike the conventional neutral red uptake assay [26], which primarily reflects overall cell viability through an absorbance-based measurement, the retention assay specifically evaluates vacuolar capacity to maintain dye accumulation under antifungal stress, employing fluorescence to enhance sensitivity. Quantification of neutral red retention showed a marked reduction in mangiferin-treated cells, while microscopy revealed swollen vacuoles, diminished vacuolar fluorescence, and cytosolic dye leakage, collectively demonstrating compromised vacuolar homeostasis.

By simultaneously impairing the cell wall and vacuolar function, mangiferin acts through dual mechanisms distinct from conventional antifungals that typically target a single pathway, such as ergosterol or β-glucan synthesis. This multitarget action may reduce the likelihood of resistance development in *C. albicans*. Previous studies reported that mangiferin enhances the antifungal activity of caspofungin by interfering with polyamine accumulation [17] and potentiates the efficacy of fluconazole against resistant isolates [18]. The present findings extend these observations and provide evidence that mangiferin compromises fungal viability by disrupting both cell wall biosynthesis and vacuolar homeostasis, highlighting its potential as a multitarget antifungal agent.

## Figures and Tables

**Table 1:** Minimum inhibitory concentrations (MICs) of mangiferin against *Candida* species.

	MIC (µg/ml)	
	Mangiferin	Amphotericin B
*C. albicans* SC5314	156	1
*C. krusei* ATCC 6258	32	0.5
*C. glabrata* ATCC 2001	8	1
*C. parapsilosis* ATCC 22019	64	1

MICs of mangiferin against *Candida* spp. determined by the CLSI M27-A3 broth microdilution method with resazurin as a viability indicator.

**Table 2:** Sorbitol protection assay and MICs of mangiferin against *C. albicans* SC5314.

Incubation time (h)	MIC of Mangiferin (µg/ml)	
	No sorbitol	Sorbitol
48	156	156
72	156	312

Antifungal susceptibility was assessed using a modified CLSI M27-A3 broth microdilution method with resazurin as a viability indicator, in the absence or presence of 0.8 M sorbitol.
